# 6-[6-(Pyridin-2-yl)-1,2,4,5-tetra­zin-3-yl]pyridin-3-amine monohydrate

**DOI:** 10.1107/S2056989016000608

**Published:** 2016-01-27

**Authors:** Johannes Broichhagen, Yvonne E. Klingl, Dirk Trauner, Peter Mayer

**Affiliations:** a Ludwig-Maximilians-Universität, Department, Butenandtstrasse 5–13, 81377 München, Germany

**Keywords:** crystal structure, hydrogen bonding, graph sets, asymmetric tetra­zines

## Abstract

The packing of the title compound, C_12_H_9_N_7_·H_2_O, is dominated by hydrogen bonding and π-stacking. Layers parallel to [010] are established by hydrogen bonds involving all amine donor functions and one of the water donor functions, while the remaining water donor function enables the stacking of the layers along [10-1], which is accompanied by π-stacking. In the molecule, the plane of the central tetra­zine ring forms angles of 5.33 (7) and 19.84 (8)° with the adjacent 3-amine-pyridine and pyridine rings, respectively.

## Abstract 2

The tetra­zine derivative, as well as the water mol­ecule, of C_12_H_9_N_7_·H_2_O are involved in a hydrogen-bond network accompanied by π-stacking.

## Chemical context   

*Click chemistry* is employed to label biological targets because of its highly selective reaction profile at ambient temperature in physiological media (Kolb *et al.*, 2001 ▸). Several chemical reactions can be used for this purpose. Among the most popular are alkyne–azide [3 + 2]-pericyclic reactions, and ene–tetra­zine Diels–Alder/retro-Diels–Alder (DA/rDA) reactions. If the biomolecule carries a clickable chemical unit, possibly installed by the introduction of unnatural amino acids, various label-bearing functionalities can be introduced efficiently (Hong *et al.*, 2010 ▸; Tsai *et al.*, 2015 ▸). Side-chain norbornenes have proven particularly successful as unnatural amino acids (Kaya *et al.*, 2012 ▸). They undergo a DA/rDA reaction with tetra­zines, resulting in the extrusion of nitro­gen (Kaya *et al.*, 2012 ▸; Vrabel *et al.*, 2013 ▸). This reaction exhibits fast kinetics at ambient temperatures, making it particularly useful for biological labeling. To improve biological stability, more electron-deficient 2-pyridinyl-substituted tetra­zines are employed as they display improved stability (Vrabel *et al.*, 2013 ▸). In order to decorate tetra­zines with functionalities, asymmetric bis­pyridyl tetra­zine versions with a desired label are synthesized. For instance, an amine group can be introduced that reacts with activated esters. Herein, we describe the crystal structure of such an asymmetric tetra­zine in its hydrate form, bearing pyridyl groups on each side, one of them exposing a free amine (Selvaraj & Fox, 2014 ▸).
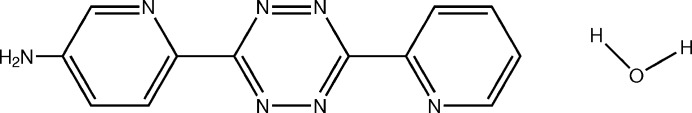


## Structural commentary   

**Figure 1:**
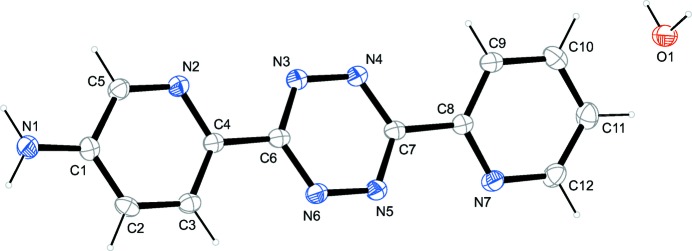
The mol­ecular structure of the title compound, showing atom labels and anisotropic displacement ellipsoids (drawn at the 50% probability level) for non-H atoms.

The asymmetric unit of the title compound, which is depicted in Fig. 1 ▸, comprises 6-[6-(pyridin-2-yl)-1,2,4,5-tetra­zin-3-yl]pyridin-3-amin (**1**) and a water mol­ecule. The three almost planar six-membered rings of **1** deviate significantly from coplanarity. The plane of the central tetra­zine ring forms angles of 5.33 (7) and 19.84 (8)° with the adjacent 3-amine-pyridine and pyridine rings, respectively. In two related structures of inversion-symmetric tetra­zines these angles are 26.41 (10)° (Liu *et al.*, 2001 ▸) and 19.71 (5)° (Klein *et al.*, 1998 ▸). The latter two terminal rings enclose an angle of 14.60 (8)° in the title compound. This observation deviates from two related structures in which the terminal pyridine rings are coplanar (Klein *et al.*, 1998 ▸; Liu *et al.*, 2001 ▸). The hydrogen atoms of the amine are almost parallel with the adjacent pyridine ring and form an angle of 120.7 (16)° with amine N1. The H—O—H angle of the water mol­ecule is 102.0 (17)°.

## Supra­molecular features   

**Figure 2:**
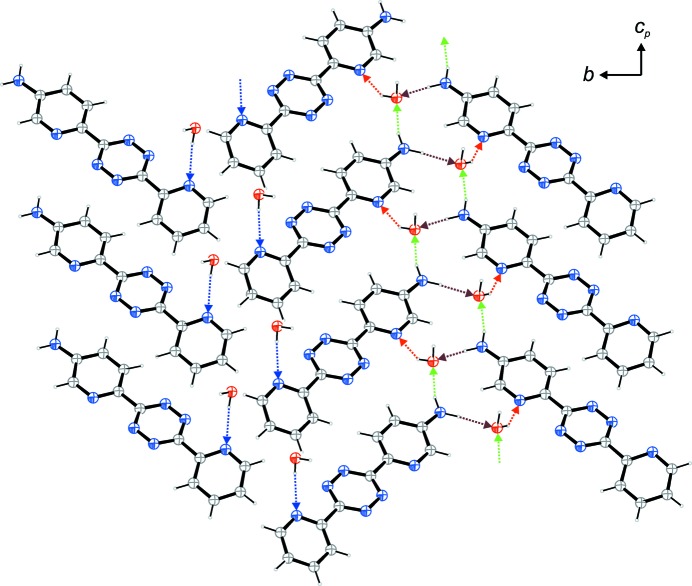
The hydrogen-bond pattern in layers viewed along [100].

Hydrogen bonding is the main feature of packing of the title compound. Both amine donor functions as well as both H atoms of the water mol­ecule are involved in hydrogen bonds with the two pyridine ring N atoms and the water mol­ecule acting as hydrogen-bond acceptors (Table 1 ▸). It shall be mentioned that the tetra­zine N5 atom is acceptor in a bifurcated hydrogen bond with donor O1. However, the donor–H–acceptor angle O1—H14⋯N5 is rather acute at 124.9 (15)° and the donor–acceptor distance rather long at 3.1934 (18) Å. Hence this hydrogen bond is not depicted in Figs. 2 ▸ and 3 ▸, and it is not considered in the following discussion of the hydrogen-bond network.

Fig. 2 ▸ shows a part of the herringbone-pattern-like layer parallel to [010] of the title compound. In that figure, the four different hydrogen bonds are shown in different colours. The region with the blue water–pyridine-N hydrogen bonds contains no amine groups. By this hydrogen bond, the layer is linked to next layer on top of it. By the other three hydrogen bonds, the moieties of the title compound form a two-dimensional network. According to graph set theory (Bernstein *et al.*, 1995 ▸; Etter *et al.*, 1990 ▸), the descriptor 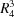
(11) can be assigned on the ternary level (three different hydrogen bonds) for the 11-membered rings formed by four hydrogen bonds involving two amine groups and two water mol­ecules (two brown, one green and one red bond). In order to outline the chains along [101] formed by two different hydrogen bonds, the graph-set descriptor 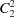
(7) may be assigned on the binary level. The seven-membered unit is formed by one N—H⋯O (green) and one O—H⋯N hydrogen bond (red).

**Figure 3:**
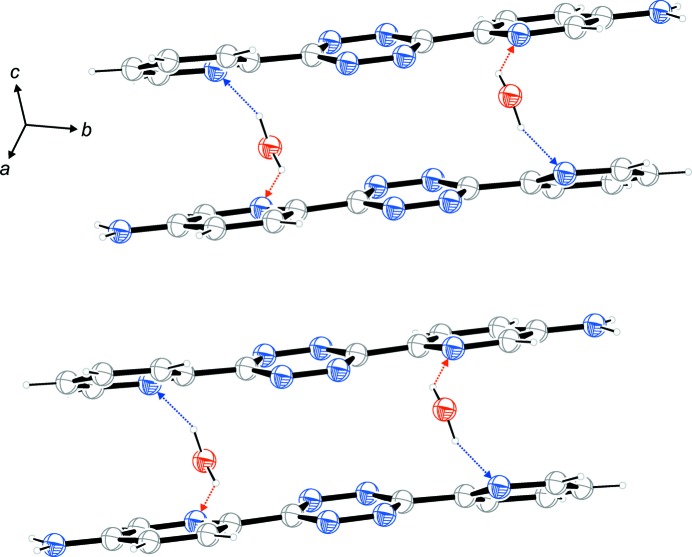
π-Stacking and hydrogen bonds in the packing of the title compound.

**Figure 4:**
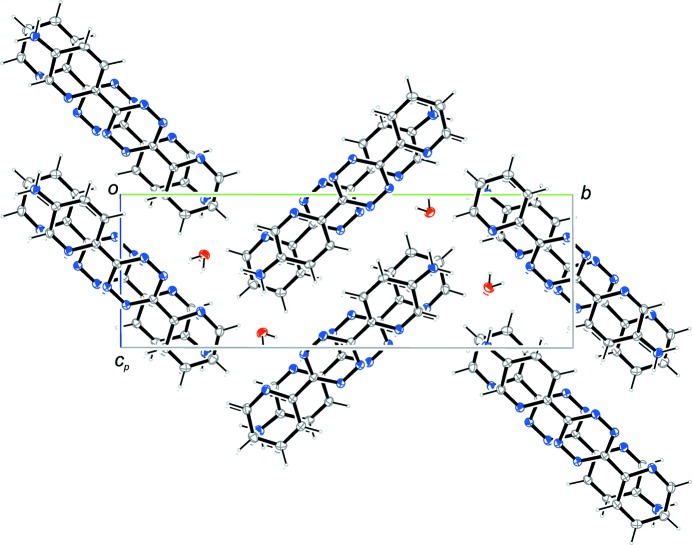
The packing of the title compound viewed along [100].

Fig. 3 ▸ shows the inter­action of stacking and hydrogen bonds. Centrosymmetric dimeric units consisting of two water and two organic mol­ecules are linked by four O—H⋯N hydrogen bonds. In terms of graph-set theory, the descriptor 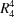
(22) can be assigned. *Within* these dimeric units, a tetra­zine ring has an adjacent tetra­zine ring – exactly parallel due to an center of inversion – with a distance of 3.5896 (9) Å between the ring centroids. Additionally, the pyridine rings have adjacent amino-pyridine rings. The dihedral angles are 14.60 (8)° with a distance of 3.7477 (9) Å between the centroids. *Between* the dimeric units, the tetra­zine ring has an adjacent amino-pyridine ring which subtends a dihedral angle of 5.33 (7)°. The distance between the ring centroids amounts to 3.6360 (9) Å. Fig. 4 ▸ shows the packing of the unit cell and gives a further impression of the herringbone pattern and the stacking.

## Synthesis and crystallization   

The title compound was synthesized according to a literature procedure (Selvaraj & Fox, 2014 ▸) and the analytical data matched that reported. Single crystals were obtained by recrystallization from hot acetone.

## Refinement   

Crystal data, data collection and structure refinement details are summarized in Table 2 ▸. C-bonded H atoms were positioned geometrically (C—H = 0.95 Å) and treated as riding on their parent atoms [*U*_iso_(H) = 1.2*U*_eq_(C)]. The coordinates of N- and O-bound hydrogen atoms were refined freely with *U*_iso_(H) = 1.2*U*_eq_(N or O).

## Supplementary Material

Crystal structure: contains datablock(s) I, global. DOI: 10.1107/S2056989016000608/zl4001sup1.cif

Structure factors: contains datablock(s) I. DOI: 10.1107/S2056989016000608/zl4001Isup2.hkl

Supporting information file. DOI: 10.1107/S2056989016000608/zl4001Isup3.cml

CCDC reference: 1446773

Additional supporting information:  crystallographic information; 3D view; checkCIF report

## Figures and Tables

**Table 1:** Hydrogen-bond geometry (Å, °)

*D*—H⋯*A*	*D*—H	H⋯*A*	*D*⋯*A*	*D*—H⋯*A*
N1—H11⋯O1^i^	0.93 (2)	2.12 (2)	3.024 (2)	166.2 (16)
N1—H12⋯O1^ii^	0.90 (2)	2.13 (2)	3.012 (2)	165.3 (16)
O1—H14⋯N5^iii^	0.87 (2)	2.614 (19)	3.1934 (18)	124.9 (15)
O1—H14⋯N7^iii^	0.87 (2)	2.12 (2)	2.9321 (18)	153.9 (17)
O1—H13⋯N2^iv^	0.88 (2)	2.19 (2)	2.9688 (18)	147.4 (16)

Symmetry codes: (i) 
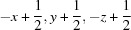
; (ii) 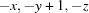; (iii) 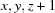; (iv) 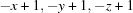.

**Table 2:** Experimental details

Crystal data	
Chemical formula	C_12_H_9_N_7_·H_2_O
*M*_r_	269.28
Crystal system, space group	Monoclinic, *P*2_1_/*n*
Temperature (K)	100
*a*, *b*, *c* (Å)	7.5488 (4), 21.4944 (14), 7.8936 (5)
β (°)	111.7170 (19)
*V* (Å^3^)	1189.88 (13)
*Z*	4
Radiation type	Mo *K*α
μ (mm^−1^)	0.11
Crystal size (mm)	0.13 × 0.08 × 0.02

Data collection	
Diffractometer	Bruker D8 Venture TXS
Absorption correction	Multi-scan (*SADABS*; Bruker, 2015 ▸)
*T*_min_, *T*_max_	0.924, 0.958
No. of measured, independent and observed [*I* > 2σ(*I*)] reflections	20441, 2186, 1751
*R*_int_	0.046
(sin θ/λ)_max_ (Å^−1^)	0.603

Refinement	
*R*[*F* ^2^ > 2σ(*F* ^2^)], *wR*(*F* ^2^), *S*	0.038, 0.101, 1.06
No. of reflections	2186
No. of parameters	193
H-atom treatment	H atoms treated by a mixture of independent and constrained refinement
Δρ_max_, Δρ_min_ (e Å^−3^)	0.28, −0.18

Computer programs: *APEX3* and *SAINT* (Bruker, 2015 ▸), *SIR97* (Altomare *et al.*, 1999 ▸), *SHELXL2014* (Sheldrick, 2015 ▸), *ORTEPIII* (Burnett & Johnson, 1996 ▸) and *PLATON* (Spek, 2009 ▸).

## supplementary crystallographic information

### Crystal data

**Table:**

C_12_H_9_N_7_·H_2_O	*F*(000) = 560
*M**_r_* = 269.28	*D*_x_ = 1.503 Mg m^−^^3^
Monoclinic, *P*2_1_/*n*	Mo *K*α radiation, λ = 0.71073 Å
*a* = 7.5488 (4) Å	Cell parameters from 4888 reflections
*b* = 21.4944 (14) Å	θ = 2.9–25.3°
*c* = 7.8936 (5) Å	µ = 0.11 mm^−^^1^
β = 111.7170 (19)°	*T* = 100 K
*V* = 1189.88 (13) Å^3^	Platelet, red
*Z* = 4	0.13 × 0.08 × 0.02 mm

### Data collection

**Table:**

Bruker D8 Venture TXS diffractometer	2186 independent reflections
Radiation source: rotating anode (TXS)	1751 reflections with *I* > 2σ(*I*)
Detector resolution: 10.4167 pixels mm^-1^	*R*_int_ = 0.046
mix of phi and ω scans	θ_max_ = 25.4°, θ_min_ = 2.9°
Absorption correction: multi-scan (*SADABS*; Bruker, 2015)	*h* = −9→9
*T*_min_ = 0.924, *T*_max_ = 0.958	*k* = −25→25
20441 measured reflections	*l* = −9→9

### Refinement

**Table:**

Refinement on *F*^2^	0 restraints
Least-squares matrix: full	Hydrogen site location: mixed
*R*[*F*^2^ > 2σ(*F*^2^)] = 0.038	H atoms treated by a mixture of independent and constrained refinement
*wR*(*F*^2^) = 0.101	*w* = 1/[σ^2^(*F*_o_^2^) + (0.0506*P*)^2^ + 0.4194*P*] where *P* = (*F*_o_^2^ + 2*F*_c_^2^)/3
*S* = 1.06	(Δ/σ)_max_ < 0.001
2186 reflections	Δρ_max_ = 0.28 e Å^−^^3^
193 parameters	Δρ_min_ = −0.18 e Å^−^^3^

### Special details

**Table:**

Geometry. All e.s.d.'s (except the e.s.d. in the dihedral angle between two l.s. planes) are estimated using the full covariance matrix. The cell e.s.d.'s are taken into account individually in the estimation of e.s.d.'s in distances, angles and torsion angles; correlations between e.s.d.'s in cell parameters are only used when they are defined by crystal symmetry. An approximate (isotropic) treatment of cell e.s.d.'s is used for estimating e.s.d.'s involving l.s. planes.

### Fractional atomic coordinates and isotropic or equivalent isotropic displacement parameters (Å^2^)

**Table:**

	*x*	*y*	*z*	*U*_iso_*/*U*_eq_	
C1	−0.1210 (2)	0.64392 (7)	−0.4086 (2)	0.0188 (4)	
C2	−0.1550 (2)	0.58094 (8)	−0.4525 (2)	0.0205 (4)	
H2	−0.2516	0.5687	−0.5642	0.025*	
C3	−0.0470 (2)	0.53676 (7)	−0.3322 (2)	0.0191 (4)	
H3	−0.0695	0.4938	−0.3604	0.023*	
C4	0.0953 (2)	0.55506 (7)	−0.1693 (2)	0.0165 (3)	
C5	0.0242 (2)	0.65788 (7)	−0.2380 (2)	0.0209 (4)	
H5	0.0473	0.7004	−0.2043	0.025*	
C6	0.2153 (2)	0.50924 (7)	−0.0406 (2)	0.0167 (3)	
C7	0.4210 (2)	0.42767 (7)	0.1870 (2)	0.0166 (3)	
C8	0.5358 (2)	0.38179 (7)	0.3232 (2)	0.0174 (3)	
C9	0.6399 (2)	0.40029 (8)	0.5007 (2)	0.0214 (4)	
H9	0.6403	0.4426	0.5356	0.026*	
C10	0.7430 (2)	0.35636 (8)	0.6260 (2)	0.0253 (4)	
H10	0.8166	0.3679	0.7480	0.030*	
C11	0.7368 (2)	0.29540 (8)	0.5699 (2)	0.0256 (4)	
H11A	0.8039	0.2639	0.6533	0.031*	
C12	0.6312 (2)	0.28102 (8)	0.3901 (2)	0.0242 (4)	
H12A	0.6301	0.2390	0.3523	0.029*	
N1	−0.2190 (2)	0.69044 (7)	−0.5185 (2)	0.0253 (4)	
H11	−0.180 (3)	0.7312 (10)	−0.487 (2)	0.030*	
H12	−0.310 (3)	0.6815 (9)	−0.628 (3)	0.030*	
N2	0.12937 (18)	0.61569 (6)	−0.12284 (18)	0.0199 (3)	
N3	0.36169 (19)	0.53003 (6)	0.10630 (17)	0.0198 (3)	
N4	0.46638 (19)	0.48774 (6)	0.22296 (18)	0.0199 (3)	
N5	0.27906 (18)	0.40634 (6)	0.03676 (17)	0.0193 (3)	
N6	0.17398 (18)	0.44830 (6)	−0.07911 (17)	0.0202 (3)	
N7	0.53069 (19)	0.32257 (6)	0.26624 (18)	0.0210 (3)	
O1	0.52583 (18)	0.31449 (6)	0.89374 (16)	0.0253 (3)	
H14	0.488 (3)	0.3167 (9)	0.985 (3)	0.030*	
H13	0.612 (3)	0.3439 (9)	0.920 (3)	0.030*	

### Atomic displacement parameters (Å^2^)

**Table:**

	*U*^11^	*U*^22^	*U*^33^	*U*^12^	*U*^13^	*U*^23^
C1	0.0160 (8)	0.0223 (8)	0.0203 (8)	−0.0006 (6)	0.0094 (6)	0.0027 (7)
C2	0.0157 (8)	0.0278 (9)	0.0170 (8)	−0.0026 (7)	0.0048 (6)	−0.0036 (7)
C3	0.0194 (8)	0.0192 (8)	0.0207 (9)	−0.0017 (7)	0.0096 (7)	−0.0030 (7)
C4	0.0166 (8)	0.0176 (8)	0.0179 (8)	−0.0026 (6)	0.0094 (6)	−0.0019 (6)
C5	0.0205 (8)	0.0184 (8)	0.0228 (9)	−0.0020 (7)	0.0070 (7)	−0.0006 (7)
C6	0.0170 (8)	0.0189 (8)	0.0177 (8)	−0.0032 (6)	0.0104 (6)	−0.0033 (6)
C7	0.0170 (8)	0.0185 (8)	0.0174 (8)	−0.0016 (6)	0.0102 (6)	−0.0032 (6)
C8	0.0164 (8)	0.0184 (8)	0.0192 (8)	−0.0013 (6)	0.0087 (6)	−0.0011 (6)
C9	0.0226 (9)	0.0200 (8)	0.0213 (9)	−0.0024 (7)	0.0079 (7)	−0.0039 (7)
C10	0.0230 (9)	0.0313 (10)	0.0186 (8)	−0.0008 (7)	0.0044 (7)	−0.0002 (7)
C11	0.0202 (9)	0.0258 (9)	0.0275 (9)	0.0022 (7)	0.0051 (7)	0.0056 (7)
C12	0.0230 (9)	0.0177 (9)	0.0296 (10)	0.0027 (7)	0.0072 (7)	0.0007 (7)
N1	0.0241 (8)	0.0223 (8)	0.0224 (8)	−0.0018 (6)	0.0004 (6)	0.0027 (6)
N2	0.0202 (7)	0.0179 (7)	0.0201 (7)	−0.0020 (6)	0.0059 (6)	−0.0002 (6)
N3	0.0211 (7)	0.0179 (7)	0.0192 (7)	−0.0005 (6)	0.0060 (6)	−0.0008 (6)
N4	0.0221 (7)	0.0167 (7)	0.0195 (7)	−0.0005 (6)	0.0063 (6)	−0.0010 (6)
N5	0.0202 (7)	0.0175 (7)	0.0191 (7)	0.0004 (5)	0.0060 (6)	−0.0007 (5)
N6	0.0212 (7)	0.0177 (7)	0.0208 (7)	−0.0015 (6)	0.0068 (6)	−0.0020 (6)
N7	0.0215 (7)	0.0179 (7)	0.0226 (7)	−0.0005 (6)	0.0071 (6)	−0.0021 (6)
O1	0.0287 (7)	0.0252 (7)	0.0220 (6)	−0.0055 (5)	0.0093 (5)	−0.0040 (5)

### Geometric parameters (Å, º)

**Table:**

C1—N1	1.351 (2)	C8—N7	1.346 (2)
C1—C2	1.397 (2)	C8—C9	1.387 (2)
C1—C5	1.419 (2)	C9—C10	1.380 (2)
C2—C3	1.376 (2)	C9—H9	0.9500
C2—H2	0.9500	C10—C11	1.378 (2)
C3—C4	1.393 (2)	C10—H10	0.9500
C3—H3	0.9500	C11—C12	1.382 (2)
C4—N2	1.352 (2)	C11—H11A	0.9500
C4—C6	1.464 (2)	C12—N7	1.334 (2)
C5—N2	1.321 (2)	C12—H12A	0.9500
C5—H5	0.9500	N1—H11	0.93 (2)
C6—N3	1.348 (2)	N1—H12	0.90 (2)
C6—N6	1.355 (2)	N3—N4	1.3268 (18)
C7—N4	1.339 (2)	N5—N6	1.3201 (18)
C7—N5	1.351 (2)	O1—H14	0.87 (2)
C7—C8	1.480 (2)	O1—H13	0.88 (2)
			
N1—C1—C2	123.47 (15)	C9—C8—C7	120.23 (14)
N1—C1—C5	120.00 (15)	C10—C9—C8	119.07 (15)
C2—C1—C5	116.53 (14)	C10—C9—H9	120.5
C3—C2—C1	119.33 (14)	C8—C9—H9	120.5
C3—C2—H2	120.3	C11—C10—C9	118.52 (15)
C1—C2—H2	120.3	C11—C10—H10	120.7
C2—C3—C4	119.98 (15)	C9—C10—H10	120.7
C2—C3—H3	120.0	C10—C11—C12	118.72 (16)
C4—C3—H3	120.0	C10—C11—H11A	120.6
N2—C4—C3	121.78 (14)	C12—C11—H11A	120.6
N2—C4—C6	116.96 (13)	N7—C12—C11	123.96 (15)
C3—C4—C6	121.26 (14)	N7—C12—H12A	118.0
N2—C5—C1	124.37 (15)	C11—C12—H12A	118.0
N2—C5—H5	117.8	C1—N1—H11	118.9 (11)
C1—C5—H5	117.8	C1—N1—H12	119.8 (12)
N3—C6—N6	124.14 (14)	H11—N1—H12	120.7 (16)
N3—C6—C4	118.29 (14)	C5—N2—C4	117.99 (14)
N6—C6—C4	117.56 (14)	N4—N3—C6	117.25 (13)
N4—C7—N5	124.82 (14)	N3—N4—C7	118.28 (13)
N4—C7—C8	116.98 (14)	N6—N5—C7	117.03 (13)
N5—C7—C8	118.21 (13)	N5—N6—C6	118.40 (13)
N7—C8—C9	122.97 (15)	C12—N7—C8	116.74 (14)
N7—C8—C7	116.79 (14)	H14—O1—H13	102.0 (17)
			
N1—C1—C2—C3	179.62 (15)	C9—C10—C11—C12	1.4 (2)
C5—C1—C2—C3	−0.8 (2)	C10—C11—C12—N7	−1.3 (3)
C1—C2—C3—C4	−0.4 (2)	C1—C5—N2—C4	−0.8 (2)
C2—C3—C4—N2	1.2 (2)	C3—C4—N2—C5	−0.6 (2)
C2—C3—C4—C6	−178.61 (14)	C6—C4—N2—C5	179.24 (13)
N1—C1—C5—N2	−178.91 (15)	N6—C6—N3—N4	−2.6 (2)
C2—C1—C5—N2	1.5 (2)	C4—C6—N3—N4	178.76 (12)
N2—C4—C6—N3	−5.9 (2)	C6—N3—N4—C7	0.4 (2)
C3—C4—C6—N3	173.94 (13)	N5—C7—N4—N3	2.2 (2)
N2—C4—C6—N6	175.42 (13)	C8—C7—N4—N3	−177.71 (13)
C3—C4—C6—N6	−4.8 (2)	N4—C7—N5—N6	−2.5 (2)
N4—C7—C8—N7	−160.90 (13)	C8—C7—N5—N6	177.39 (13)
N5—C7—C8—N7	19.2 (2)	C7—N5—N6—C6	0.2 (2)
N4—C7—C8—C9	20.0 (2)	N3—C6—N6—N5	2.3 (2)
N5—C7—C8—C9	−159.94 (14)	C4—C6—N6—N5	−179.07 (13)
N7—C8—C9—C10	−0.2 (2)	C11—C12—N7—C8	0.4 (2)
C7—C8—C9—C10	178.84 (14)	C9—C8—N7—C12	0.4 (2)
C8—C9—C10—C11	−0.7 (2)	C7—C8—N7—C12	−178.73 (13)

### Hydrogen-bond geometry (Å, º)

**Table:**

*D*—H···*A*	*D*—H	H···*A*	*D*···*A*	*D*—H···*A*
N1—H11···O1^i^	0.93 (2)	2.12 (2)	3.024 (2)	166.2 (16)
N1—H12···O1^ii^	0.90 (2)	2.13 (2)	3.012 (2)	165.3 (16)
O1—H14···N5^iii^	0.87 (2)	2.614 (19)	3.1934 (18)	124.9 (15)
O1—H14···N7^iii^	0.87 (2)	2.12 (2)	2.9321 (18)	153.9 (17)
O1—H13···N2^iv^	0.88 (2)	2.19 (2)	2.9688 (18)	147.4 (16)

Symmetry codes: (i) −*x*+1/2, *y*+1/2, −*z*+1/2; (ii) −*x*, −*y*+1, −*z*; (iii) *x*, *y*, *z*+1; (iv) −*x*+1, −*y*+1, −*z*+1.
